# A Tale of Many Cities: Universal Patterns in Human Urban Mobility

**DOI:** 10.1371/journal.pone.0037027

**Published:** 2012-05-29

**Authors:** Anastasios Noulas, Salvatore Scellato, Renaud Lambiotte, Massimiliano Pontil, Cecilia Mascolo

**Affiliations:** 1 Computer Laboratory, University of Cambridge, Cambridge, United Kingdom; 2 Department of Mathematics, University of Namur, Namur, Belgium; 3 Department of Computer Science, University College London, London, United Kingdom; University of Oxford, United Kingdom

## Abstract

The advent of geographic online social networks such as Foursquare, where users voluntarily signal their current location, opens the door to powerful studies on human movement. In particular the fine granularity of the location data, with GPS accuracy down to 10 meters, and the worldwide scale of Foursquare adoption are unprecedented. In this paper we study urban mobility patterns of people in several metropolitan cities around the globe by analyzing a large set of Foursquare users. Surprisingly, while there are variations in human movement in different cities, our analysis shows that those are predominantly due to different distributions of places across different urban environments. Moreover, a universal law for human mobility is identified, which isolates as a key component the rank-distance, factoring in the number of places between origin and destination, rather than pure physical distance, as considered in some previous works. Building on our findings, we also show how a rank-based movement model accurately captures real human movements in different cities.

## Introduction

Since the seminal works of Ravenstein [1], the movement of people in space has been an active subject of research in the social and geographical sciences. It has been shown in almost every quantitative study and described in a broad range of models that a close relationship exists between mobility and distance. People do not move randomly in space, as we know from our daily lives. Human movements exhibit instead high levels of regularity and tend to be hindered by geographical distance. The origin of this dependence of mobility on distance, and the formulation of quantitative laws explaining human mobility remains, however, an open question, the answer of which would lead to many applications, e.g. improve engineered systems such as cloud computing and location-based recommendations [2]–[5], enhance research in social networks [6]–[9] and yield insight into a variety of important societal issues, such as urban planning and epidemiology [10]–[12].

In classical studies, two related but diverging viewpoints have emerged. The first camp argues that mobility is directly deterred by the costs (in time and energy) associated to physical distance. Inspired by Newton's law of gravity, the flow of individuals is predicted to decrease with the physical distance between two locations, typically as a power-law of distance [13]–[15]. Besides distance, more complex versions of gravity models may also consider a parameter that captures the “mass” of the starting point and the destination of a trip. In this case, usually the population of an area is used as a proxy to quantify it. These so-called “gravity-models” have a long tradition in quantitative geography and urban planning and have been used to model a wide variety of social systems, e.g. human migration [16], inter-city communication [17] and traffic flows [18]. The second camp argues instead that there is no direct relation between mobility and distance, and that distance is a surrogate for the effect of *intervening opportunities* [19]. The migration from origin to destination is assumed to depend on the number of opportunities closer than this destination. A person thus tends to search for destinations where to satisfy the needs giving rise to its journey, and the absolute value of their distance is irrelevant. Only their ranking matters. Displacements are thus driven by the spatial distribution of places of interest, and thus by the response to opportunities rather than by transport impedance as in gravity models. The first camp appears to have been favoured by practitioners on the grounds of computational ease [20], despite the fact that several statistical studies have shown that the concept of intervening opportunities is better at explaining a broad range of mobility data [21]–[25].

This long-standing debate is of particular interest in view of the recent revival of empirical research on human mobility. Contrary to traditional works, where researchers have relied on surveys, small-scale observations or aggregate data, recent research has taken advantage of the advent of pervasive technologies in order to uncover trajectories of millions of individuals with unprecedented resolution and to search for universal mobility patterns, such to feed quantitative modelling. Interestingly, those works have all focused on the probabilistic nature of movements in terms of physical distance. As for gravity models, this viewpoint finds its roots in Physics, in the theory of anomalous diffusion. It tends to concentrate on the distributions of displacements as a function of geographic distance. Recent studies suggest the existence of a universal power-law distribution 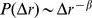, observed for instance in cell tower data of humans carrying mobile phones 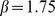 [26] or in the movements of “Where is George” dollar bills 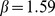 [27]. This universality is, however, in contradiction with observations that displacements strongly depend on where they take place. For instance, a study of hundreds of thousands of cell phones in Los Angeles and New York demonstrate different characteristic trip lengths in the two cities [28]. This observation suggests either the absence of universal patterns in human mobility or the fact that physical distance is not a proper variable to express it.

In this work, we address this problem by focusing on human mobility patterns in a large number of cities across the world. More precisely, we aim at answering the following question: “Do people move in a substantially different way in different cities or, rather, do movements exhibit universal traits across disparate urban centers?”. To do so, we take advantage of the advent of mobile location-based social services accessed via GPS-enabled smartphones, for which fine granularity data about human movements is becoming available. Moreover, the worldwide adoption of these tools implies that the scale of the datasets is planetary. Exploiting data collected from public *check-ins* made by users of the most popular location-based social network, Foursquare [29], we study the movements of 925,030 users around the globe over a period of about six months, and study the movements across 5 million places in 34 metropolitan cities that span four continents and eleven countries.

After discussing how at larger distances we are able to reproduce previous results of [26] and [27], we also offer new insights on some of the important questions about human urban mobility across a variety of cities. We first confirm that mobility, when measured as a function of distance, does not exhibit universal patterns. The striking element of our analysis is that we observe a universal behavior in all cities when measured with the right variable. We discover that the probability of transiting from one place to another is inversely proportional to a power of their *rank*, that is, the number of intervening opportunities between them. This universality is remarkable as it is observed despite cultural, organizational and national differences. This finding comes into agreement with the social networking parallel which suggests that the probability of a friendship between two individuals is inversely proportional to the number of friends between them [30], and depends only indirectly on physical distance. More importantly, our analysis is in favour of the concept of intervening opportunities rather than gravity models, thus suggesting that trip making is not explicitly dependent on physical distance but on the accessibility of resources satisfying the objective of the trip. Individuals thus differ from random walkers in exploring physical space because of the motives driving their mobility.

Our findings are confirmed with a series of simulations verifying the hypothesis that the place density is the driving force of urban movement. By using only information about the distribution of places of a city as input and by coupling this with a rank-based mobility preference we are able to reproduce the actual distribution of movements observed in real data. These results open new directions for future research and may positively impact many practical systems and application that are centered on mobile location-based services.

## Results

### Urban Movements and Power-laws

We draw our analysis upon a dataset collected from the largest Location-based Social Network, Foursquare [29]. The dataset features 35,289,629 movements of 925,030 users across 4,960,496 places collected during six months in 2010. Foursquare places or venues are geo-tagged Web entities which correspond to real venues in the physical world, e.g. coffee shops, airport terminals or libraries, and which are associated to precise geographic coordinates, expressed with latitude and longitude. In this context a movement is the indication of presence at a place that a user gives through the Foursquare system. In the present work we focus on the 34 cities with the highest number of check-ins in the dataset. The reader can view summary statistics for all cities we have experimented with in Table 1.

**Table 1:** Summary of city statistics.

City Name	Movements	Places	Density (Places/ 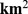 )	Area ( 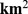 )	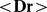 ( 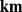 )
Amsterdam	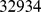	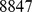	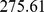	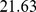	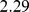
Atlanta	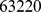	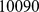	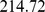	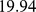	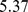
[gray].9 Austin	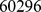	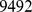	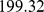	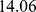	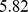
Bangkok	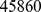	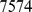	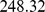	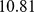	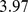
[gray].9 Boston	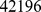	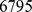	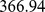	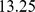	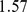
Chicago	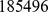	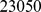	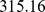	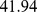	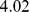
[gray].9 Columbus	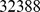	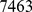	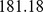	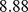	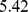
Dallas	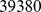	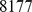	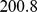	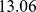	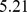
[gray].9 Denver	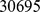	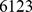	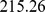	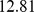	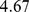
Houston	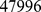	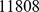	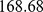	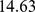	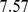
[gray].9 Indianapolis	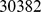	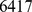	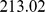	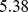	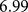
Kuala Lumpur	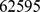	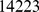	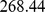	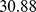	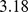
[gray].9 Las Vegas	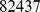	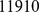	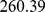	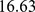	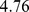
London	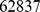	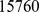	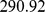	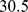	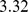
[gray].9 Los Angeles	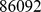	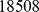	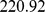	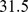	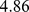
Milwaukee	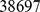	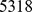	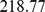	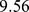	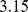
[gray].9 Minneapolis	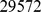	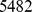	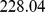	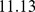	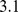
New York	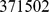	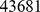	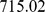	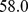	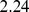
[gray].9 Orlando	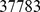	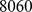	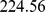	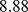	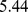
Paris	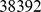	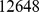	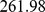	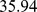	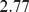
[gray].9 Philadelphia	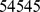	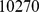	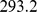	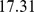	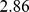
Phoenix	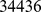	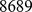	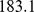	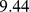	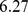
[gray].9 Portland	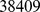	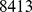	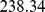	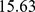	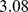
Rio de Janeiro	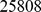	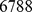	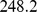	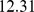	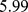
[gray].9 San Antonio	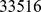	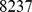	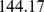	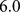	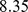
San Diego	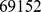	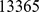	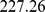	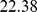	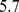
[gray].9 San Francisco	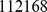	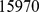	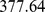	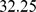	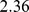
Santiago	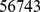	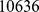	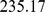	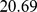	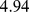
[gray].9 Seattle	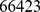	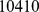	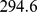	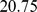	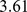
Seoul	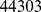	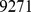	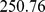	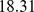	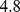
[gray].9 Singapore	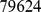	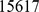	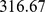	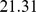	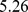
São Paulo	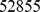	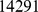	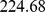	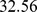	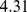
[gray].9 Toronto	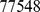	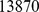	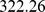	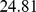	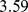
Washington	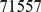	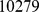	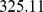	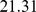	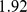

**Figure 1:**
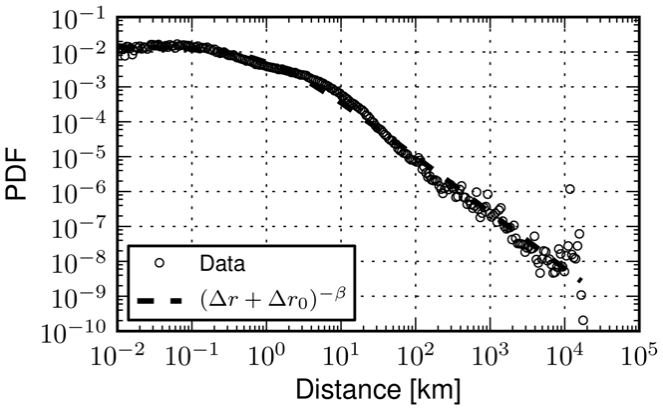
Global movements. The probability density function (PDF) of human displacements as seen through 35 million location broadcasts (check-ins) across the planet. The power-law fit features an exponent 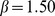 and a threshold 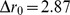 confirming previous works on human mobility data. The spatial granularity offered by GPS data allows for the inspection of human movements at very small distances, whereas the global reach of Foursquare reveals the full tail of the planetary distribution of human movements.

**Figure 2:**
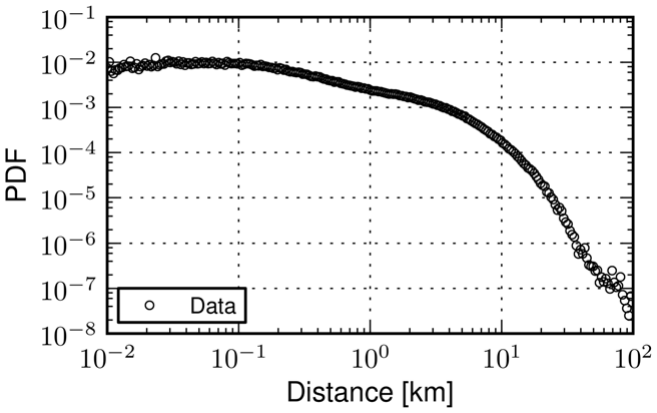
Urban movements. The probability density function (PDF) of human displacements in cities (intracity). For two successive location broadcasts (check-ins) a sample is included if the locations involved in the transition belong to the same city. Approximately 10 million of those transitions have been measured. The poor power-law fit of the data (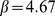, 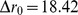) suggests that the distribution of intracity displacements can not be fully described by a power law. Short transitions which correspond to a large portion of the movements distribution are not captured by such process.

In order to confirm the large scale results reported in [26], [27], we have computed the distribution of human displacements in our dataset (Figure 1): we observe that the distribution is well approximated by a power law with exponent 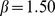 and a threshold 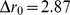 (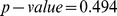). This is almost identical to the value of the exponent calculated for the dollar bills movement (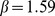) [27] and very proximate to the 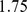 estimated from cellphones calls analysis of human mobility [26]. With respect to these datasets, we note that the Foursquare dataset is planetary, as it contains movements at distances up to 20,000 kilometres (we measure all distances using the great-circle distance between points on the planet). On the other extreme, small distances of the order of tens of meters can also be approximated thanks to the fine granularity of GPS technology employed by mobile phones running these geographic social network applications. Indeed, we find that the probability of moving up to 100 meters is uniform, a trend that has also been shown in [27] for a distance threshold 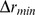. Each transition in the dataset happens between two well defined venues, with data specifying the city they belong to. We exploit this information to define when a transition is urban, that is, when both start and end points are located within the same city. Figure 2 depicts the probability density function of the about 10 million displacements within cities across the globe. We note that a power-law fit does not accurately capture the distribution. First of all, a large fraction of the distribution exhibits an initial flat trend; then, only for values larger than 10 km the tail of distribution decays, albeit with a very large exponent which does not suggest a power-law tail. Overall, power-laws tend to be captured across many orders of magnitude, whereas this is not true in the case of urban movements. The estimated parameter values via Maximum Likelihood are 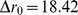 and exponent 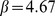 (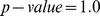). A detailed description of used methods can be found in the final section of this manuscript.

### Movements across cities

**Figure 3:**
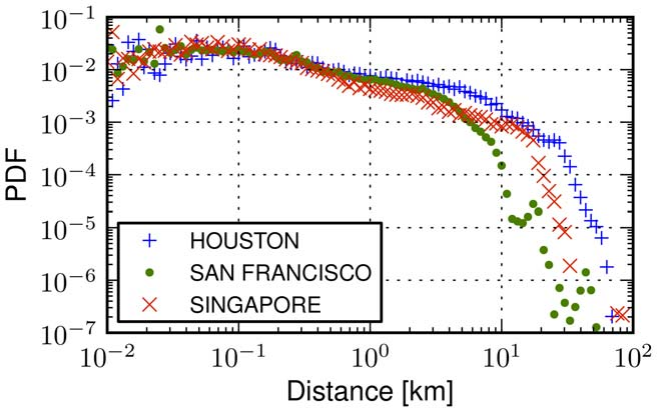
Urban movement heterogeneities. The probability density function (PDF) of human displacements in three cities: Houston, San Francisco and Singapore (for 47, 112 and 79 thousand transitions, respectively). Common trends are observed, e.g., the probability of a jump steadily decreases after the distance threshold of 100 meters, but the shapes of the distributions vary from city to city, suggesting either that human movements do not exhibit universal patterns across cities or that distance is not the appropriate variable to model them.

Since the distribution of urban human movements cannot be approximated with a power law distribution nor with a physically relevant functional relation, how can we represent displacements of people in a city more appropriately? We start by comparing human movements across different cities. In Figure 3, we plot the distribution of human displacements for Houston, San Francisco and Singapore noting that similar patterns have been observed across all cases we have considered in the experiments. The shapes of the distributions, albeit different, exhibit similarities suggesting the existence of a common underlying process that seems to characterize human movements in urban environments. There is an almost uniform probability of traveling in the first 100 meters, that is followed by a decreasing trend between 100 meters and a distance threshold 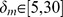 km, where we detect an abrupt cutoff in the probability of observing a human transition. The threshold 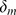 could be due to the reach of the *borders* of a city, where maximum distances emerge.

While the distributions exhibit similar trends in different cities, scales and functional relation may differ, thus suggesting that human mobility vary from city to city. For example, while comparing Houston and San Francisco (see Figure 3), different thresholds 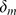 are observed. Moreover, the probability densities can vary across distance ranges. For instance, it is more probable to have a transition in the range 300 meters and 5 kilometers in San Francisco than in Singapore, but the opposite is true beyond 5 kilometers. This difference could be attributed to many potential factors, ranging from geographic ones such as area size, density of a city, to differences in infrastructures such as transportation and services or even socio-cultural variations across cities. In the following paragraphs we present a formal analysis that allow to dissect these heterogeneities.

### The importance of place density

**Figure 4:**
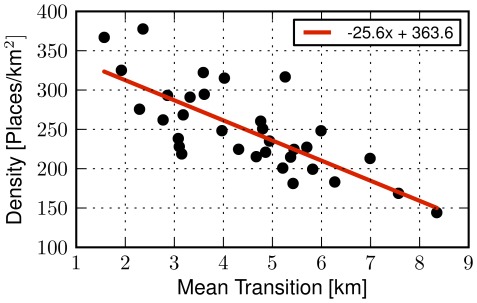
City place densities and mean movement lengths. Scatter plot of the density of a city, defined as the number of places per square kilometer, versus its mean human transition in kilometers. Each datapoint corresponds to a city, while the red line is a fit that highlights the relationship of the two variables (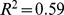). A longer mean transition corresponds to the expectation of a sparser urban environment, indicating that the number of available places per area unit could have an impact on human urban travel.

**Figure 5:**
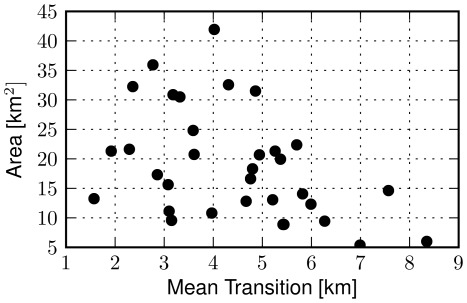
City area sizes and mean movement lengths. Scatter plot of the area of a city, measured in square kilometers, versus its mean human transition in kilometers. Unlike place density, the area of a city does not seem strongly related to the mean length of its transitions (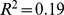). To measure the area of a city we have segmented the spatial plane around its geographic midpoint in squares of size 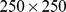 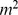. The area of a city has been defined as the sum area of all squares that feature at least five places.

Inspired by Stouffer's theory of intervening opportunities [19] which suggests that *the number of persons traveling a given distance is directly proportional to the number of opportunities at that distance and inversely proportional to the number of intervening opportunities*, we explore to what extend the density of places in a city is related to the human displacements within it. First, we define the density of a city in the Foursquare dataset by applying a grid onto each city using squares of area size equal 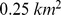 and filtering out those grid areas which feature less than five Foursquare venues. Then the density is equal to the number of places per square 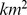 averaged across the grid. As a next step, we plot the place density of a city, as computed with our check-in data, against the average distance of displacements observed in a number of cities. In Figure 4 one observes that the average distance of human movements is *inversely proportional* to the city's density. Hence, in a very dense metropolis, like New York, there is a higher expectation of shorter movements. We have measured a coefficient of determination 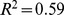. Intuitively, this correlation suggests that while distance is a cost factor taken into account by humans, the range of available places at a given distance is also important. This availability of places may relate to the availability of resources while performing daily activities and movements: if no super markets are around, longer movements might be more probable in order to find supplies. As a next step, we explore whether the geographic area size covered by a city affects human mobility by plotting the average transition in a city versus its area size (see Figure 5). Our data indicates no apparent linear relationship, with a low correlation 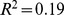, thus indicating that density is a more informative measure.

**Figure 6:**
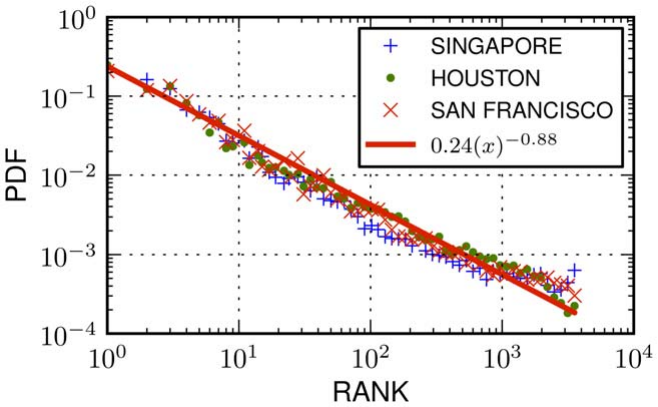
Rank distributions in three cities. (a) Probability density function (PDF) of rank values for three cities (Houston, Singapore and San Francisco). Our methodology to measure the rank distribution is the following: for each transition between two places 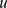 and 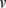, we measure 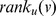 defined as the number of places that are geographically closer to 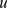 than 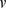. We observe that the distributions of the three cities collapse to a single line, which suggests that universal laws can be formulated in terms of the rank variable. The observation confirms the hypothesis that human movements are driven by the density of the geographic environment rather than the exact distance cost of our travels. A least squares fit (red line) underlines the decreasing trend of the probability of a jump as the rank of a places increases.

**Figure 7:**
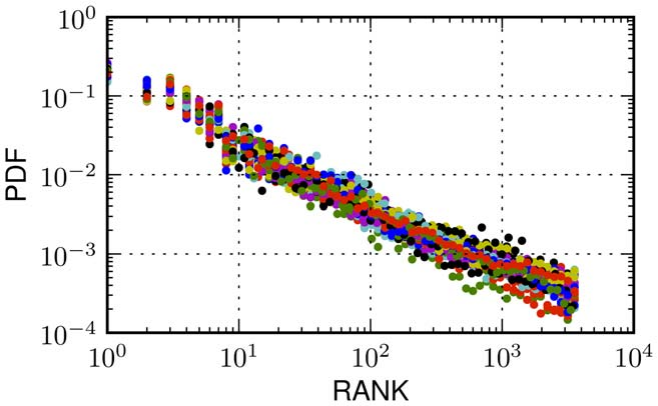
Rank distributions in urban environments. Superimposition of the probability density functions (PDF) of rank values the thirty-four cities analyzed in the Foursquare dataset. A decreasing trend for the probability of a jump at a place as its rank value increases is common. The trend remains stable despite the large number of plotted cities and their potential differences with respect to a number of variables such us number of places, number of displacements, area size, density or other cultural, national or organizational ones.

To shed further light on the hypothesis that density is a decisive factor in human mobility, for every movement between a pair of places in a city we sample the rank value of it. The rank for each transition between two places 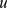 and 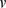 is the number of places 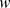 that are closer in terms of distance to 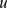 than 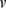 is. Formally: 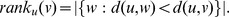 The rank between two places has the important property to be invariant in scaled versions of a city, where the relative positions of the places is preserved but the absolute distances dilated. In Figure 6 we plot for the three cities the rank values observed for each displacement. The fit of the rank densities on a log-log plot, shows that the rank distribution follows linear trend similar to that of a power-law distribution. This observation suggests that the probability of moving to a place decays when the number of places nearer than a potential destination increases. Moreover, the ranks of all cities collapse on the same line despite the variations in the probability densities of human displacements. We have fit the rank distribution for the thirty-four cities under investigation and have measured an exponent 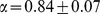. This is indicative of a universal pattern across cities where density of settlements is the driving factor of human mobility. We superimpose the distribution of ranks for all cities in Figure 7.

Interestingly enough, a parallel of this finding can be drawn with the results in [30], where it is found that the probability of observing a user's friend at a certain distance in a geographic social network is inversely proportional to the number of people geographically closer to the user.

### Modelling urban mobility

The universal mobility behaviour emerging across cities paves the way to a new model of movement in urban environments. Given a set of places 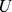 in a city, the probability of moving from place 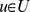 to a place 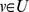 is formally defined as
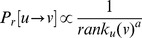
where
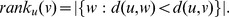



In addition to the *rank-distance* model presented above, we have adopted a gravity-based model of human urban movement. In this context such model should incorporate two factors. On the one hand, the deterrence affect of distance in movement, and on the other hand, the attractiveness of places due to a gravitational force. The former factor is captured in a straightforward way by measuring the geographic distance, 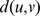, between two places 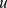 and 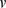. Next, we need to quantify the *gravitational mass* of a place 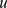. To achieve this, we measure the number of nearby settlements assuming that the denser the area that surrounds a place, the higher its attractiveness. That has required the use of an additional parameter 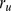, which corresponds to the radius of the circle centered on the geographic position of place 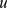. We can now define the mass 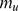 of 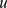, simply by enumerating the number of places that fall within the circle's surface. The probability of a transition between two places 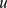 and 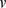 in the gravity-based model is set to be proportional to the product of the places' masses and inversely proportional to their geographic distance. Formally
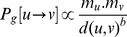



**Figure 8:**
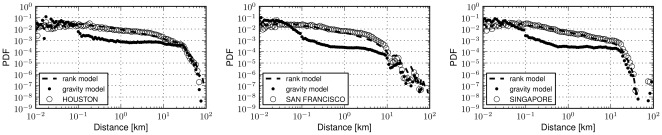
Fitting urban movements. Probability Density Functions (PDF) of human movements and corresponding fits with the rank-distance and gravity models in three cities (Houston, San Francisco and Singapore). In the rank-distance model the probability of transiting from a place 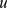 to a place 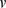 in a city, only depends on the rank value of 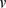 with respect to 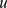. In the case of the gravity model, the deterrence affect of distance is co-integrated with a mass based attractiveness of a place 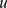. The associated mass, 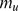, has been defined according to the number of neighboring places. The parameters for the depicted fit of the gravity model are 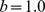 and 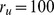 meters. The places of a city employed for the simulation experiments where those observed in the Foursquare dataset, hence while the rank-based model is the same for all cities the underlying spatial distribution of places may vary. Excellent fits are observed for all cities analyzed. It is interesting to note that the model is able to reproduce even minor anomalies, such as the case of San Francisco where we have ‘jumps’ in the probability of a movement at 20 and 40 kilometers.

We run agent based simulation experiments (see detailed description in Methods Section) where agents transit from one place to another according to the probabilities defined by the two models above. Averaging the output of the probability of movements by considering all possible places of a city as potential starting points for our agents, we present the human displacements resulting from the model in Figure 8: as shown, despite the simplicity of the rank model, this is able to capture with very high accuracy the real human displacements in a city. On the contrary, the gravity model does not present a desirable fit, since small distances are overestimated. A potential explanation for this behaviour could be given by the fact that in urban environments most settlements are positioned in a central, highly dense, core of a city. In this case, not rare in an urban context, the probability of a transition to a proximate place may rise dramatically when considering a gravity model, as density reaches a maximum and geographic distances are minimized.

**Figure 9:**
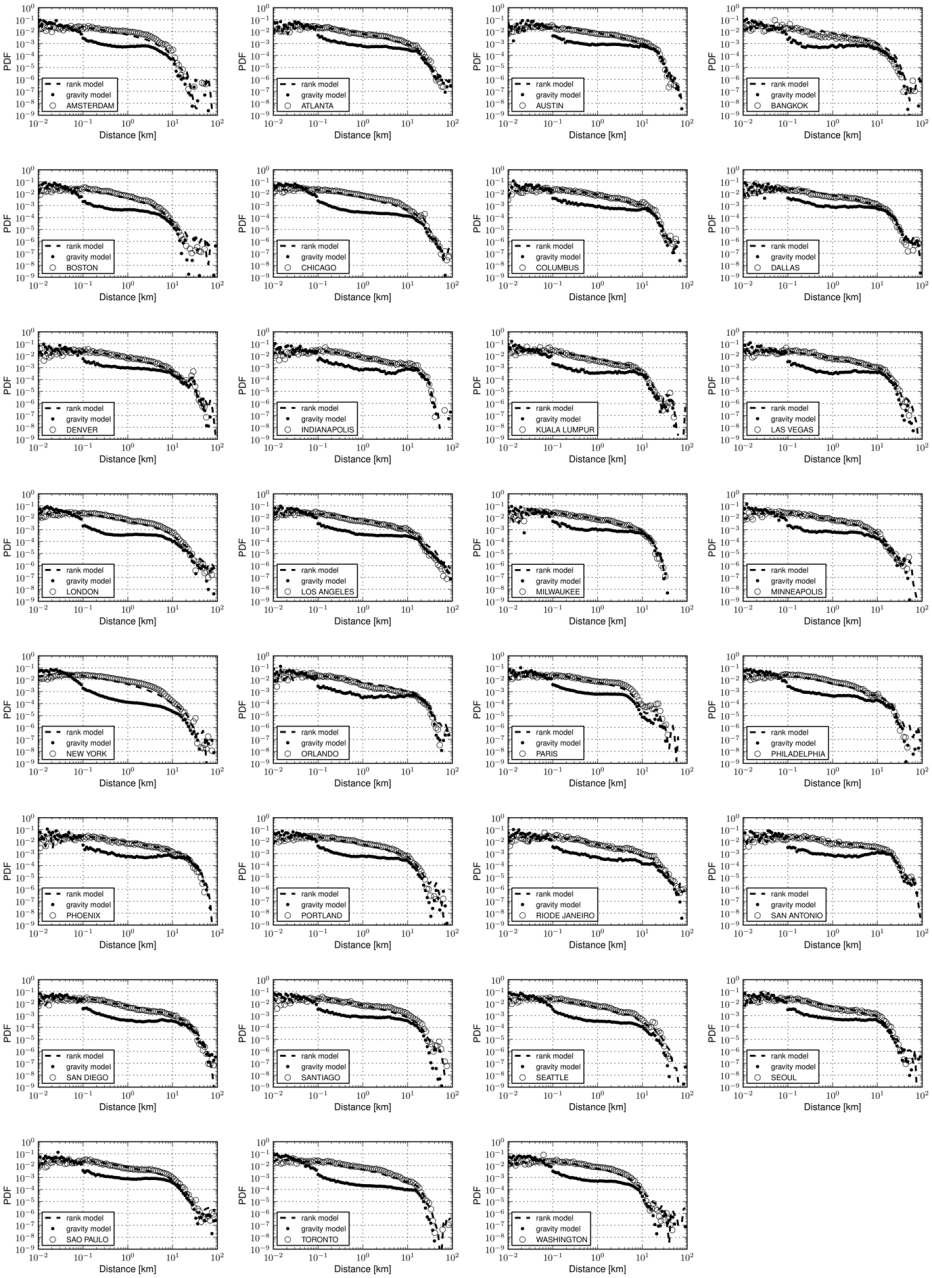
Fitting urban movements for all cities in the Foursquare dataset. The dominance of the rank-distance model over the gravity case extends to the rest of the cities (34 in total) we have experimented with in the Foursquare dataset. The results depicted here correspond to the gravity model with parameters 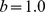 and 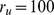 meters, whereas in the case of the rank-distance model an exponent 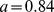 has been used to simulate movement in all cities and corresponds to the empirical average of the exponents resulting from the fit of the rank value distributions.

Besides comparing the performance of the two models in the task of fitting the empirical distributions of human movement, it is worth discussing their parameterization too. In the case of the *rank-distance* model, a common parameter 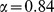 has been set for the simulations of all cities. That is the empirical average observed by fitting the distributions of the rank values observed in cities as depicted in Figure 7. Given the small standard deviations observed across cities, it is remarkable to observe that it would be sufficient to observe movements in one city and fit accurately the transitions of others, provided we have knowledge on the geographic position of their settlements. On the other hand, the identification of the parameters for the gravity model was a more complex task. Initially, we had to choose a radius 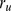 to define the mass 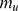 of a place 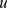. While this would have been easier to perform if we were considering movement across countries, or across cities, by considering for instance the size of their populations, it is much harder to define a similar geographic or organizational scope within a city. In our experiments we tested exhaustively 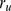 values ranging from 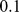 to 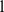 kilometers. Equally, selecting an exponent 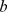 to control the effect of distance in movements required again a brute-force exploration of values (we have experimented for values within the range 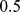 to 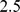). We note that our aim is not to exclude the possibility that more complex gravity models could be devised achieving potentially better fits of urban movement. Nonetheless, in light of the evidence that our experiments have provided, the use of a rank-distance variable qualifies better for the division of a universal urban mobility model. Moreover, it is worth noting that the rank model does not take into account other parameters such as individual heterogeneity patterns [26] or temporal ones [27] that have been studied in the past in the context of human mobility and yet it offers very accurate matching of the human traces of our dataset. Plots with the performance of the models for all thirty four cities that we have evaluated can be found in Figure 9.

### Controlling urban geography

**Figure 10:**
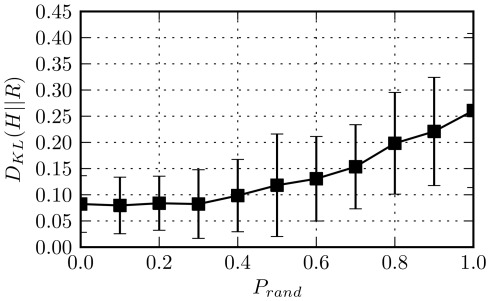
Geographic distribution of places in cities. Gaussian kernel density estimation (KDE) applied on the spatial distribution of places in three cities (Houston, San Francisco and Singapore). Each dark point corresponds to a venue observed in the Foursquare dataset encoded in terms of longitude and latitude values. The output of the KDE is visualised with a thermal map. A principal core of high density is observed in the three cities, but point-wise density and spatial distribution patterns may differ. The rank-based model can cope with those heterogeneities as it accounts for the relative density for a given pair of places 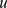 and 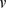.

**Figure 11:**
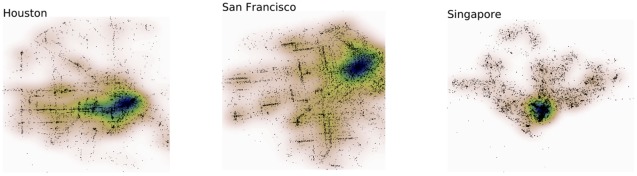
Probability density function (PDF) of observing two randomly selected places at a distance 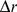
 **in a city.** We have enumerated 11808, 15970, 15617 unique venues for Houston, San Francisco and Singapore respectively. The probability is increasing with 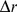, as expected in two dimensions before falling due to finite size effect. It is interesting to note that the probability for two randomly selected places to be the origin and destination of a jump monotonically decreases with distance (see SI).

This analysis provides empirical evidence that while human displacements across cities may differ, these variations are mainly due to the spatial distribution of places in a city instead of other potential factors such as social-cultural or cognitive ones. Indeed, the agent based simulations are run with the same rules and parameters in each city, except for the set of places 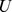 that is taken from the empirical dataset. The variation across the spatial organization of cities is illustrated in Figure 10, where we plot thermal maps of the density of places within cities and in Figure 11, where we plot the probability density function that two random places are at a distance 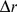. Both figures highlight large heterogeneities in the distribution of places across cities and have provoked us to examine further how the geography of a city, encoded through the longitudinal positions of its settlements, impacts human mobility. Could we then *alter* the spatial distribution of settlements in a city and quantify the affect of this process in human movement?

**Figure 12:**
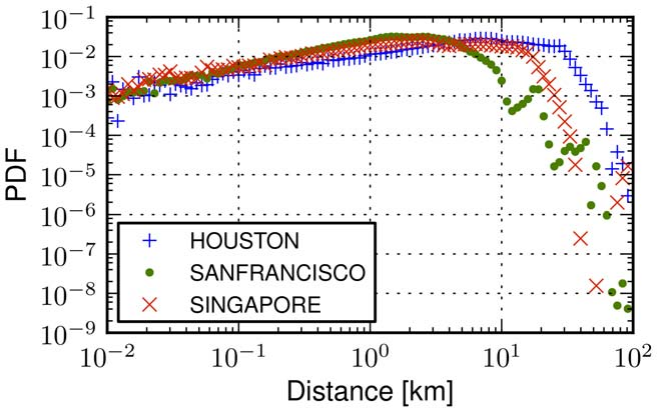
Effect of place coordinate randomization on the performance of the rank-distance model. On the y-axis we present the KL-divergence, 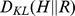, between the empirically observed distribution of displacements in a city 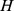 and 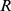 which is the one obtained by the *rank-distance* model. On the x-axis the probability of randomization, 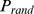, is depicted. In order to randomize the spatial distribution of places in a city, we iterate through the associated set of places 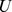 and the coordinates of a place 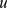, 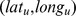 are randomized with probability 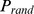. A new pair of coordinates, 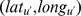, is assigned uniformly and within a pre-specified range, where 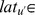 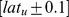 and 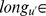 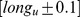. 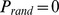 corresponds to the case that the original distribution of displacements within a city is maintained, whereas the opposite extreme where 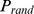 equals 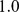 means that all places have been randomized. The errors bars correspond to standard deviations across cities.

The methodology we have put forward to demonstrate this is based on the spatial randomization of places, 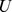, of a city. We do so by iterating through all places in 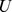 and randomizing the coordinates, 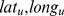, of a place 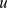 with probability 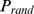. A new pair of latitude and longitude coordinates is elected, 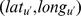, by considering a uniform sample in a predefined range, where 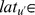 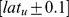 and 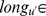 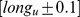. In Figure 12, we present the Kullback–Leibler Divergence (KL-Divergence), 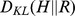, between the empirically observed distribution of human displacements, 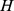, and the distribution 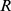 obtained by the *rank-distance* model for different values 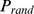. The KL-Divergence [31] is a non-symmetric measure of the difference between two probability distributions and is formally defined here as
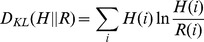
The reader may observe that as the probability of randomizing the position of a place increases, the quality of the fit attained by the *rank-distance* model on average drops. This observation becomes statistically significant only for 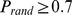. We note that any alternative randomization process which, instead, preserves the relative density between all pairs of places would not have an impact with regards to the performance of the model on the original set of places 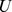 (or 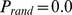 equivalently). That is expected as the probability of a transition in the *rank-distance* model is dependent exclusively on this factor. Overall, this analysis highlights the impact of geography, as expressed through the spatial distribution of places, on human movements, and confirms at a large-scale the seminal analysis of Stouffer [19] who studied how the spatial distribution of places and employment opportunities in the city of Cleveland affected the migration movements of families.

## Discussion

The empirical data on human movements provided by Foursquare and other location-based services allows for unprecedented analysis both in terms of scale and the information we have about the details of human movements. The former means that mobility patterns in different parts of the world can be analyzed and compared surpassing cultural, national or other organizational borders. The latter is achieved through better location specification technologies such as GPS-enabled smartphones, but also with novel online services that allow users to layout content on the geographical plane such as the existence of places and semantic information about those. As those technologies advance our understanding on human behavior can only become deeper.

In this article, we have focused on human mobility in a large number of metropolitan cities around the world to perform an empirical validation of past theories on the driving factors of human movements. As we have shown, Stouffer's [19] theory of intervening opportunities appears to be a plausible explanation to the observed mobility patterns. The theory suggests that the distance covered by humans is determined by the number of opportunities (i.e., places) within that distance, and not by the distance itself. This behaviour is confirmed in our data where we observed that physical distance does not allow for the formulation of universal rules for human mobility, whereas a universal pattern emerges across all cities when movements are analyzed through their respective rank values: the probability of a transition to a destination place is inversely proportional to the relative rank of it, raised to a power 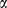, with respect to a starting geographical point. Moreover, 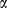 presents minor variations from city to city.

We believe that our approach opens avenues of quantitative exploration of human mobility, with several applications in urban planning and ICT. The identification of rank as an appropriate variable for the deterrence of human mobility is in itself an important observation, as it is expected to lead to more reliable measurements in systems where the density of opportunities is not uniform, e.g. in a majority of real-world systems. The realization of universal properties in cities around the globe also goes along the line of recent research [32], [33] on urban dynamics and organization, where cities have been shown to be scaled versions of each other, despite their cultural and historical differences. Contrary to previous observations where size is the major determinant of many socio-economical characteristics, however, density and spatial distribution are the important factors for mobility. Moreover, the richness of the dataset naturally opens up new research directions, such as the identification of the needs and motives driving human movements, and the calibration of the contact rate, e.g. density- vs frequency-dependent, in epidemiological models [34]. The current study also shares the interests in determining the universal laws governing human mobility and migration patterns with [35]. We concentrate on modelling movement at the city scale, using the distribution of places in cities while the radiation model presented in [35] exploits population densities to model larger scale mobility patterns across states or municipalities. Finally, we note that there may be a strong demographic bias in the community of Foursquare users. While this is an inherent characteristic of many telecommunication services and corresponding datasets, it is encouraging that the analysis and models developed in the context of the present work demonstrate strong similarities across multiple urban centers and different countries. Moreover, these data appear to exhibit properties similar to those in mobile phone cellular data [8], [9].

## Materials and Methods

The mobility dataset used in this work is comprised from *check-ins* made by Foursquare users and become publicly available through Twitter's Streaming API. The collection process lasted from the 27th of May 2010 until the 3rd of November of the same year. During this period we have observed 35,289,629 *check-ins* from 925,030 unique users over 4,960,496 venues. In addition, *locality* information together with exact GPS geo-coordinates for each venue has become available through the Foursquare website allowing us to associate a given venue with a city. By considering only consecutive *check-ins* that take place within the same city we have extracted almost 10 million *intracity* movements analysed in Figure 2. Detailed statistics including the number of check-ins and venues in each city can be found in Table 1.

We have employed the methods detailed in [36] to apply goodness-of-fit tests on the Probability Density Functions of global and urban transitions observed in Figures 1 and 2. In particular, we have measured the corresponding 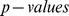 using the Kolmogorov-Smirnov test by generating 1000 synthetic distributions, while the Maximum-Likelihood Estimation technique has been used to estimate the parameters of the power-laws. Exceptionally, we have resorted to a *least squares* based optimization to measure the exponent 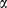 of the rank values shown in Figure 6, because a power-law distribution is not, strictly speaking, well-defined for exponents smaller than 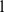. However we are confident of the values estimated due to the excellent movement fits produced during our simulations.

We now describe the rank-based model we have devised with the aim to fit human movements. Our aim is to calculate the displacement probability distribution over a given city, which is described by a set of places 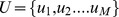. We measure the pairwise transition probability from a starting place 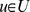 to a destination place 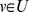 as
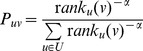
where, recall that 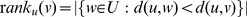 and we use the convention that 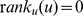 for every 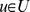. The above configuration takes into account all places in the city away from 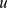 and suggests a probabilistic setting that the sum of the probabilities of transition to any destination place is equal to 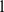.

Elaborating further, we define the probability of observing a movement of length 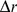 away from an initial place 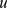 as
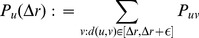
where 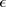 is some prescribed “resolution” parameter. We can now measure the probability of observing a transition of length within 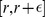 considering an arbitrary starting place 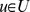 as
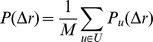
.

We note that the parameter 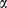 of the model has been set equal to 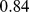 in all cases. This is the empirically calculated average of the rank value distributions, observed across the cities of the Foursquare dataset. The parameter 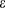 has been set by binning the x-axis logarithmically using 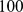 bins in the range 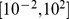. To obtain the Probability Density Functions (PDF) shown in the figures, we have divided 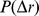 with the size of each bin, that is 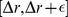.
