# Correction: A Tale of Many Cities: Universal Patterns in Human Urban Mobility

**DOI:** 10.1371/annotation/ca85bf7a-7922-47d5-8bfb-bcdf25af8c72

**Published:** 2012-09-18

**Authors:** Anastasios Noulas, Salvatore Scellato, Renaud Lambiotte, Massimiliano Pontil, Cecilia Mascolo

The last three figures were misplaced. The figure published as Figure 10 is actually Figure 12. The figure published as Figure 11 is actually Figure 10. the figure published as Figure 12 is actually Figure 11. The legends are in the correct locations.

There were errors in the References list in the online version of the article. The References list in the PDF is correct. 

